# Is thyroid nodule volume predictive for malignancy?

**DOI:** 10.20945/2359-3997000000113

**Published:** 2019-03-18

**Authors:** Nagihan Bestepe, Didem Ozdemir, Husniye Baser, Berna Ogmen, Nuran Sungu, Mehmet Kilic, Reyhan Ersoy, Bekir Cakir

**Affiliations:** 1 Ankara Ataturk Educational and Research Hospital Department of Endocrinology and Metabolism Ankara Turkey Ankara Ataturk Educational and Research Hospital, Department of Endocrinology and Metabolism, Ankara, Turkey; 2 Yildirim Beyazit University Yildirim Beyazit University School of Medicine Department of Endocrinology and Metabolism Ankara Turkey Yildirim Beyazit University, School of Medicine, Department of Endocrinology and Metabolism, Ankara, Turkey; 3 Yildirim Beyazit University Yildirim Beyazit University School of Medicine Department of Pathology Ankara Turkey Yildirim Beyazit University, School of Medicine, Department of Pathology, Ankara, Turkey; 4 Yildirim Beyazit University Yildirim Beyazit University School of Medicine Department of General Surgery Ankara Turkey Yildirim Beyazit University, School of Medicine, Department of General Surgery, Ankara, Turkey

**Keywords:** Thyroid nodule, nodule volume, malignancy

## Abstract

**Objective:**

We aimed to determine the roles of preoperative thyroid nodule diameter and volume in the prediction of malignancy.

**Subjects and methods:**

The medical records of patients who underwent thyroidectomy between January 2007 and December 2014 were reviewed. The nodule diameters were grouped as < 1 cm, 1-1.9 cm, 2-3.9 cm and ≥ 4 cm, and volume was grouped as > 5 cm^3^, 5-9.9 cm^3^ and > 10 cm^3^. ROC (Receiver Operating Characteristic) curve analysis was performed to find the optimal cutoff value of diameter and volume that can predict malignancy.

**Results:**

There were 5561 thyroid nodules in 2463 patients. Five hundred and forty (9.7%) nodules were < 1 cm, 2,413 (43.4%) were 1-1.9 cm, 1,600 (28.8%) were 2-3.9 cm and 1,008 (18.1%) were ≥ 4 cm. Malignancy rates were 25.6%,10.6%, 9.7% and 8.5% in nodules < 1 cm, 1-1.9 cm, 2-3.9 cm and ≥ 4 cm, respectively. When classified according to volume, 3,664 (65.9%) nodules were < 5 cm^3^, 594 (10.7%) were 5-9.9 cm^3^ and 1,303 (23.4%) were ≥ 10 cm^3^. The malignancy rates were 12.7%, 11.4% and 7.8% for the nodules < 5 cm^3^, 5-9.9 cm^3^ and ≥ 10 cm^3^, respectively (p < 0.001). In ROC curve analysis, an optimal cutoff value for diameter or volume that can predict malignancy in all thyroid nodules or nodules ≥ 4 cm could not be determined.

**Conclusion:**

In this surgical series, malignancy risk did not increase with increasing nodule diameter or volume. Although the volume of malignant nodules ≥ 4 cm was higher than that of benign nodules ≥ 4 cm, there was no optimal cutoff value. The diameter or volume of the nodule cannot be used to predict malignancy or decide on surgical resection.

## INTRODUCTION

Thyroid cancer is the most common type of endocrine malignancy. According to a survey conducted in 2012, it constitutes 2.1% of all malignancies worldwide, with an ever-increasing incidence for both genders (1). This apparent increase might be related to more common use of screening techniques nowadays. Thyroid ultrasonography (US) is more commonly and widely used in patients with thyroid dysfunctions or for nodules incidentally detected during neck or thorax imaging or carotid US performed for different indications. This approach results in earlier detection and treatment of thyroid cancers in spite of the increasing incidence (2).

While the prevalence of thyroid nodules determined by palpation is 5% for females and 1% for males, these rates increase to 19%-68% when high-resolution US is used (3–6). It is critical to detect and evaluate these nodules for possible thyroid cancer because 5%-15% of all thyroid nodules are histopathologically malignant (7). High-resolution US is the preferred imaging method for thyroid nodules, and fine needle aspiration biopsy (FNAB) is considered the gold standard method for the discrimination of benign and malignant nodules preoperatively. Diagnostic samples are obtained in 89%-95% of FNAB procedures, and 55%-74% are reported as definitively benign, while 2%-5% as definitively malignant. The remaining samples are categorized as cytologically indeterminate, which includes atypia of undetermined significance/follicular lesion of undetermined significance (AUS/FLUS) in 2%-18% of nodules, follicular neoplasia/suspicious for follicular neoplasia (FN/SFN) in 2%-25%, and suspicious for malignancy (SM) in 1%-6% (8–12).

Clinical features and US findings play an important role in predicting the malignancy risk of a nodule. Young age, male sex, family history of thyroid cancer and radiotherapy to the head and neck region are factors shown to be related with increased risk of thyroid cancer (13,14). Ultrasonographically, hypoechoic pattern, solid composition, presence of microcalcification, irregular margin, increased vascularity and high strain index in elastosonography are suspicious features for malignancy (15,16).

There is a strong correlation between the lesion size and malignancy potential in the majority of cancer types (17). However, the relationship between increased nodule size and thyroid cancer is still controversial. A number of studies reported an increased risk of malignancy with increasing nodule size (18–21), while many others denied an independent relationship (22–25). The largest nodule diameter was generally considered the nodule size in the majority of these studies. To our knowledge, nodule volume was not evaluated in thyroid cancer previously. In this study, we aimed to determine the role of nodule volume as a risk factor for malignancy and to compare the diagnostic values of nodule diameter and volume in the prediction of malignancy.

## SUBJECTS AND METHODS

In this retrospective study, patients who underwent total thyroidectomy and/or lobectomy between January 2007 and December 2014 in our clinic were evaluated. Operation was performed based on the decision of a committee composed of endocrinology, general surgery, nuclear medicine and pathology specialists. Local ethical committee approval was obtained in accordance with the ethical standards of the Declaration of Helsinki. Patients who had a family history of thyroid carcinoma or exposure to radiotherapy to the head and neck region were excluded, as well as the patients with incidental papillary microcarcinoma. Patients for whom there were no preoperative US findings, whose postoperative histopathological results were not available and whose preoperative US and postoperative histopathological lesions were not matched were also excluded.

For preoperative US, the Esaote color Doppler US (Model 796 FDII; MAG Technology Co. Ltd., Yung-Ho City, Taipei, Taiwan) and a superficial probe (Model LA523 13-4 5.5-12.5 MHz) were used. The anteroposterior (AP), transverse (T) and longitudinal (L) diameters of each nodule were measured by US. The volume of nodules was calculated using the ellipsoid formula (AP x T x L x π/6) (cm^3^).

Thyroid nodules were grouped into four categories (< 1 cm, 1-1.9 cm, 2-3.9 cm and ≥ 4 cm) based on the largest nodule diameter. Similarly, the volume of nodules was examined in three groups: > 5 cm^3^, 5-9.9 cm^3^ and > 10 cm^3^.

Thyroid nodules were matched with postoperative pathological lesions in accordance with the nodule localization defined in preoperative US. Histopathological results were classified as benign and malignant. Nodular hyperplasia, colloidal goiter, follicular adenoma and Hurthle cell adenoma were defined as benign thyroid lesions. Malignant nodules were subgrouped as papillary thyroid cancer (PTC), follicular/Hurthle cell carcinoma and other malignancies (medullary, anaplastic, thyroid lymphoma, metastatic tumor, etc.). The malignancy rates were calculated in the diameter and volume groups separately.

In addition, a separate analysis including only differentiated thyroid cancer was performed, and these tumors were compared with benign nodules. In this analysis, other tumors (medullary, anaplastic, thyroid lymphoma, metastatic tumor, etc.) were excluded from the malignant group.

The SPSS 24.0 software package (IBM Corp., Armonk, NY, USA) was used for statistical analysis. We presented the descriptive statistics as mean ± standard deviation and nominal variables as number of cases and percentages. A comparison between categorical variables was made using a chi-square t-test. Student's t-test for parametric variables and Fisher's exact test or the Mann-Whitney U test for nonparametric variables were used to investigate the difference between groups. A p-value lower than 0.05 was accepted as indicating statistical significance. Additionally, we performed ROC curve analysis for nodule diameter and volume in order to find an optimal cutoff value that can predict malignancy.

## RESULTS

Data of 5,561 thyroid nodules in 2,463 patients were analyzed. There were 541 (22.0%) male and 1,922 (78.0%) female patients, and the mean age of the cohort was 49.0 ± 12.4 years (range: 17-85 years). The mean nodule diameter and volume were 2.43 ± 1.63 cm (range: 0.39-12.2 cm) and 8.5 ± 17.0 cm^3^ (range: 0.007-237 cm^3^), respectively.

### Histopathological results of nodules classified according to the maximal diameter

There were 540 (9.7%) nodules < 1 cm, 2,413 (43.4%) nodules 1-1.9 cm, 1,600 (28.8%) nodules 2-3.9 cm and 1008 (18.1%) nodules ≥ 4 cm (Table 1). Malignancy rates were 25.6%, 10.6%, 9.7% and 8.5% in nodules < 1 cm, 1-1.9 cm, 2-3.9 cm and ≥ 4 cm, respectively. When the nodule group of 1-3.9 cm was evaluated separately, the malignancy rate was 10.2%. The malignancy rates were 12.0% and 8.5% for the nodules < 4 cm and ≥ 4 cm, respectively (p = 0.002).

**Table 1:** Comparison of mean diameter and volume in histopathologically benign and malignant thyroid nodules classified according to maximal diameter

		Histopathology		p
		Benign	Malignant	
**All nodules (cm)**		2.47 ± 1.62	2.08 ± 1.59	**< 0.001**
	< 1 cm (n = 540)	n = 402 (74.4%)	n = 138 (25.6%)	
	Diameter (cm)	0.86 ± 0.11	0.81 ± 0.12	**< 0.001**
	Volume (cm^3^)	0.212 ± 0.087	0.202 ± 0.090	0.289
**1-1.9 cm (n = 2,413)**		**n = 2,158 (89.4%)**	**n = 255 (10.6%)**	
	Diameter (cm)	1.4 ± 0.28	1.33 ± 0.27	**< 0.001**
	Volume (cm^3^)	0.91 ± 0.62	0.83 ± 0.56	< 0.047
**2-3.9 cm (n = 1,600)**		**n = 1,445 (%90.3)**	**n = 155 (% 9.7)**	
	Diameter (cm)	2.84 ± 0.58	2.82 ± 0.57	0.594
	Volume (cm^3^)	7.0 ± 4.6	6.6 ± 4.1	0.278
**≥ 4 cm (n = 1,008)**		**n = 922 (91.5%)**	**n = 86 (8.5%)**	
	Diameter (cm)	5.29 ± 1.48	5.46 ± 1.41	0.448
	Volume (cm^3^)	33.2 ± 26.4	42.0 ± 36.1	0.010

In the group of nodules < 1 cm, the mean diameter was 0.86 ± 0.11 cm in histopathologically benign and 0.81 ± 0.12 cm in malignant groups (p < 0.001). In this group, volumes of benign and malignant nodules were similar (Table 1). In the group of nodules 1-1.9 cm, the mean diameter was 1.4 ± 0.28 cm in histopathologically benign and 1.33 ± 0.27 cm in malignant groups (p < 0.001). In this group, volumes of benign and malignant nodules were similar. In the group of 2-3.9 cm, mean diameters and volumes were similar for benign and malignant nodules (p = 0.594 and p = 0.278, respectively). For the nodules ≥ 4 cm, the mean diameter was 5.29 ± 1.48 cm in benign and 5.46 ± 1.41 cm in malignant nodules (p = 0.448), and the mean volume of benign nodules was significantly lower than that of malignant nodules (33.2 ± 26.4 cm^3^ *vs.* 42.0 ± 36.1 cm^3^, p = 0.01).

Among 138 malignant nodules < 1 cm, 97.1% were PTC, 2.2% were follicular/Hurthle cell carcinoma and 0.7% were other types of malignancies (Table 2). There were 255 malignant nodules in the group of 1-1.9 cm. Of these nodules, 94.9% were PTC, 3.9% were follicular/Hurthle cell carcinoma and 1.2% were others. Among 155 malignant nodules of 2-3.9 cm, 85.2% were PTC, 11.6% were follicular/Hurthle cell carcinoma and 3.2% were others. Out of 86 malignant nodules ≥ 4 cm, 67.4% were PTC, 22.1% were follicular/Hurthle cell carcinoma and 10.5% were others (Table 2).

**Table 2:** Distribution of malignancy types in groups classified according to the maximal nodule diameter

Diameter	Papillary thyroid cancer	Follicular/Hurthle cell cancer	Other*	p
< 1 cm (n = 138)	134 (97.1%)	3 (2.17%)	1 (0.72%)	**< 0.001**
1-1.9 cm (n = 255)	242 (94.9%)	10 (3.9%)	3 (1.2%)	
2-3.9 cm (n = 155)	132 (85.2%)	18 (11.6%)	5 (3.2%)	
≥ 4 cm (n = 86)	58 (67.4%)	19 (22.1%)	9 (10.5%)	

* Other: medullary, anaplastic, thyroid lymphoma, metastatic tumor etc.

### Histopathological results of nodules classified according to the volume

**Figure 1:**
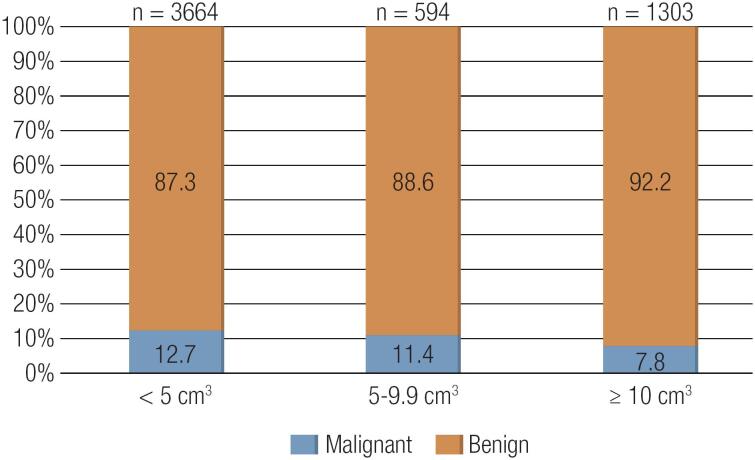
Malignancy rates of thyroid nodules classified according to volume.

There were 3,664 (65.9%) nodules < 5 cm^3^, 594 (10.7%) nodules 5-9.9 cm^3^ and 1,303 (23.4%) nodules ≥ 10 cm^3^. The malignancy rates were 12.7%, 11.4% and 7.8% for the nodules < 5 cm^3^, 5-9.9 cm^3^ and ≥ 10 cm^3^, respectively (p < 0.001) (Figure 1). When nodules were grouped as < 10 cm^3^ and ≥ 10 cm^3^, 533 (12.5%) of 4,258 nodules < 10 cm^3^ and 101 (7.8%) of 1,303 nodules ≥ 10 cm^3^ were malignant (p < 0.001).

According to the analysis including all nodules, the mean volume was 8.7 ± 1.98 cm^3^ in histopathologically benign and 7.4 ± 0.96 cm^3^ in malignant groups (p = 0.036).

In the group of nodules < 5 cm^3^, the mean volume was 1.3 ± 1.2 cm^3^ in histopathologically benign and 1.0 ± 1.1 cm^3^ in malignant groups (p = 0.007). In this group, mean diameters of benign and malignant nodules were similar (Table 3). In the 5-9.9 cm^3^ group, mean diameters and volumes were similar for benign and malignant nodules (p = 0.444 and p = 0.5, respectively).

**Table 3:** Comparison of mean diameter and volume in histopathologically benign and malignant thyroid nodules classified according to nodules volume

		Histopathology		p
		Benign	Malignant	
**All nodules (cm**^3^)		8.7 ± 1.98	7.4 ± 0.96	0.036
	**< 5 cm**^3^ **(n = 3,664)**	**n = 3,199(87.3%)**	**n = 465 (12.7%)**	
	Diameter (cm)	1.52 ± 0.53	1.32 ± 0.53	0.506
	Volume (cm^3^)	1.3 ± 1.2	1.0 ± 1.1	**0.007**
**5-9.9 cm**^3^ **(n = 594)**		**n = 526 (88.6%)**	**n = 68 (11.4%)**	
	Diameter (cm)	3.07 ± 0.5	3.14 ± 0.43	0.444
	Volume (cm^3^)	7.3 ± 1.4	7.3 ± 1.5	0.5
**≥ 10 cm**^3^ **(n = 1,303)**		**n = 1,202 (92.2%)**	**n = 101 (7.8%)**	
	Diameter (cm)	4.87 ± 1.49	5.09 ± 1.48	0.326
	Volume (cm^3^)	28.9 ± 24.4	37.0 ± 34.1	**0.003**

For the nodules ≥ 10 cm^3^, the mean diameter was 4.87 ± 1.49 cm in benign and 5.09 ± 1.48 cm in malignant nodules (p = 0.326), and the mean volume of benign nodules was significantly lower than that of malignant nodules (28.9 ± 24.4 cm^3^ *vs.* 37.0 ± 34.1 cm^3^, p = 0.003).

Rates of PTC among < 5 cm^3^, 5-9.9 cm^3^ and ≥ 10 cm^3^ malignant nodules were 94.2%, 83.8% and 71.3%, respectively (p < 0.001) (Table 4). Follicular/Hurthle cell carcinoma constituted 4.3% of malignant nodules < 5 cm^3^, 11.8% of malignant nodules 5-9.9 cm^3^ and 21.8% of malignant nodules ≥ 10 cm^3^.

**Table 4:** Distribution of malignancy types in groups classified according to the nodule volume

Volume	Papillary thyroid cancer	Follicular/Hurthle cell cancer	Other*	p
< 5 cm^3^ (n = 465)	438 (94.2%)	20 (4.3%)	7 (1.5%)	**< 0.001**
5-9.9 cm^3^ (n = 68)	57 (83.8%)	8 (11.8%)	3 (4.4%)	
≥ 10 cm^3^ (n = 101)	73 (71.3%)	22 (21.8%)	6 (5.9%)	

* Other: medullary, anaplastic, thyroid lymphoma, metastatic tumor etc.

According to the results of the analysis including only differentiated thyroid cancer (DTC), in the group of nodules < 5 cm^3^, the mean volume was 1.3 ± 1.2 cm^3^ in histopathologically benign nodules and 1.0 ± 1.1 cm^3^ in DTC (p = 0.001). In this group, mean diameters of benign nodules and DTC were similar (Table 5). In the 5-9.9 cm^3^ group, mean diameters and volumes were similar for benign nodules and DTC (p = 0.465 and p = 0.929, respectively).

**Table 5:** Comparison of mean diameter and volume in histopathologically benign thyroid nodules and differentiated thyroid cancer classified according to nodules volume

		Histopathology		p
		Benign	DTC*	
**All nodules (cm**^3^)		8.7 ± 1.98	6.9 ± 0.86	**0.012**
**< 5 cm3 (n = 3,657)**		**n = 3,199 (87.5%)**	**n = 458 (12.5%)**	
	Diameter (cm)	1.52 ± 0.53	1.32 ± 0.52	0.501
	Volume (cm^3^)	1.3 ± 1.2	1.0 ± 1.1	**0.001**
**5-9.9 cm3 (n = 591)**		**n = 526 (89%)**	**n = 65 (11%)**	
	Diameter (cm)	3.07 ± 0.5	3.12 ± 0.40	0.465
	Volume (cm^3^)	7.3 ± 1.4	7.2 ± 1.5	0.929
**≥ 10 cm3 (n = 1,297)**		**n = 1,202 (92.7%)**	**n = 95 (7.3%)**	
	Diameter (cm)	4.87 ± 1.49	5.03 ± 1.38	0.322
	Volume (cm^3^)	28.9 ± 24.4	34.4 ± 26.32	**0.031**

DTC: differentiated thyroid cancer.

For the nodules ≥ 10 cm^3^, the mean diameter was 4.87 ± 1.49 cm in benign and 5.03 ± 1.38 cm in DTC (p = 0.322), and the mean volume of benign nodules was significantly lower than that of DTC (28.9 ± 24.4 cm^3^ *vs.* 34.4 ± 26.32 cm^3^, p = 0.031).

### ROC curve analysis

**Figure 2:**
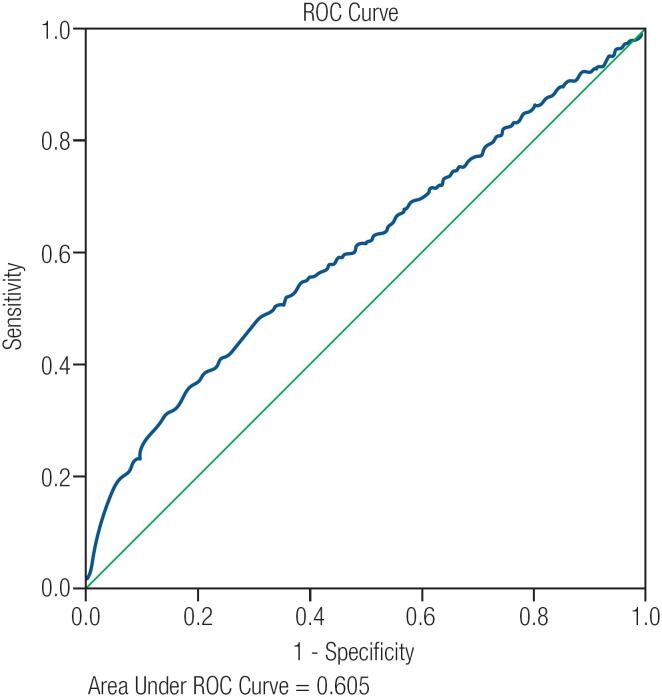
ROC Curve analysis for maximal nodule diameter.

**Figure 3:**
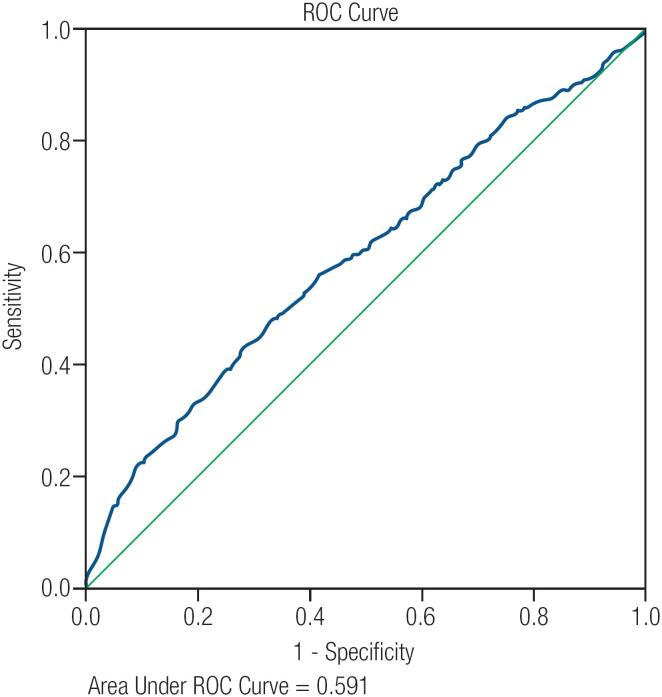
ROC Curve analysis for nodule volume.

ROC curve analysis showed that neither the maximal diameter nor the volume of the nodule was predictive of malignancy (Figures 2 and 3). In other words, there was no optimal cutoff value for either diameter or volume that could be used to predict the malignancy of thyroid nodules. Additionally, we could not find any cutoff value for volume that could help to diagnose malignancy in nodules ≥ 4 cm.

## DISCUSSION

In this retrospective study, the malignancy rates were determined based on the diameter and volume of nodules in patients who had undergone total thyroidectomy or lobectomy. We evaluated the relationship between the thyroid nodule size and risk of malignancy and tried to determine whether the nodule diameter or volume can be used to discriminate benign and malignant nodules. There was no apparent malignancy risk increment for large nodules. In addition, neither the nodule diameter nor the volume was an independent variable for malignancy.

Most previous studies reported controversial results on the relationship between the nodule size and malignancy risk. Thyroid nodules ≥ 4.0 cm are generally referred for surgical removal, even if they have benign FNAB results, demonstrate no structural impingement upon surrounding neck structures and have no cosmetic problems. This approach is most probably based on previous studies that reported increased malignancy and false-negative rates for large thyroid nodules. Furthermore, the fact that the size is an important predictor of malignancy for most other tissue types, such as the lung or adrenocortical gland, might influence this preference. The results reported in previous studies are still controversial and cannot indicate whether the size of the nodule should be considered when making a decision about whether or not to perform surgery for thyroid nodules. In a recent meta-analysis of 10 studies, Shin and cols. investigated the relationship between nodule size and postoperative histological diagnosis (24). Three studies showed a higher risk of malignancy in nodules ≥ 4.0 compared to nodules < 4.0 cm (18,20,26). However, five studies reported no significant increase in the risk of malignancy in the case of larger nodule size (22–24,27,28). Kamran and cols. examined 7,348 nodules in 4,955 patients who were operated on over the span of 14 years. The malignancy rates were 10.5% and 13% for the nodules < 2 cm and > 2 cm, respectively. They suggested a size-risk threshold of 2 cm (20). In the present study, malignancy rate was found to be 25.6% for nodules < 1 cm, 10.2% for nodules 1-3.9 cm and 8.5% for nodules ≥ 4.0 cm, showing that the risk of malignancy decreases with increasing nodule size. We have discussed these results in our previous study (29). In summary, we have indicated that this finding might be related to the selection criteria of our study, which included an operative cohort. In addition, we performed FNAB in < 1.0 cm nodules when there were clinical indicators or suspicious US features as defined by the 2009 American Thyroid Association guidelines (7) and operated on these small nodules with higher suspicion of malignancy.

In the present study, we also observed that the malignancy rate was lowest for nodules ≥ 10 cm^3^, whereas it was the highest for nodules < 5 cm^3^, showing that there is an inverse relationship between the nodule volume and the risk of malignancy, similar to the relationship between nodule diameter and malignancy. The mean volumes were similar for benign and malignant nodules.

When grouped according to nodule diameter, histopathologically malignant nodules < 1 cm had a significantly lower diameter compared to benign nodules < 1 cm, while nodule volumes were similar. For nodules 2-3.9 cm, both the mean diameters and the mean volumes were similar in benign and malignant nodules. However, in the group of nodules ≥ 4 cm, histopathologically benign and malignant nodules had similar mean diameters, whereas malignant nodules exhibited significantly larger volume compared to benign ones. When grouping was made according to volume, the mean diameters of histopathologically benign and malignant nodules ≥ 10 cm^3^ were similar, although mean volume was higher in malignant ones. Almost all studies in the literature regarding the relationship between nodule size and malignancy have considered the nodule diameter as the size parameter. The present study is the first to evaluate the nodule volume as the size parameter, which is calculated by using all three diameters of a nodule. We demonstrated that histopathologically malignant nodules ≥ 4 cm had significantly higher volume than benign nodules ≥ 4 cm. This result suggests that in nodules with the largest diameter ≥ 4 cm, malignancy risk increases with increasing volume.

Because the medullary and anaplastic carcinomas are symptomatic earlier, they are detected earlier. Therefore, this situation may cause a bias in the analysis. Thus, we performed a separate analysis including only DTC; however, we could not find any significant difference from the analysis including all thyroid cancers.

An important explanation for this might be higher risk of malignancy in round compared to oval nodules. However, based on the results of ROC curve analysis, there was no independent relationship between nodule volume and malignancy in ≥ 4 cm nodules, and therefore, no clear cutoff value could be determined that can predict malignancy. Similarly, considering all nodules, ROC curve analysis showed that neither nodule diameter nor nodule volume was a good predictor of malignancy (AUC = 0.60 and AUC = 0.59, respectively), and there was no clear cutoff value for nodule size that can predict malignancy.

Kamran and cols. reported that the proportion of PTC decreased while follicular/Hurthle carcinoma increased as nodules enlarged (20). In the present study, we obtained similar results, showing that the rate of follicular/Hurthle cell carcinoma increased significantly as the nodule diameter increased. This was also true regarding the nodule volume. The rate of PTC, which is the most common type of thyroid cancer, was highest in the group with smaller nodule diameter and volume. In other words, larger malignant nodules had higher rates of follicular and Hurthle cell carcinoma or other rare types of malignancy. These results suggest that the diagnosis of PTC is achieved at an early stage and does not originate from a benign lesion. On the contrary, the increasing rate of follicular and Hurthle cell carcinoma in larger nodules can be attributed to the fact that nodule growth increases the risk of secondary genomic mutations, which transform a benign nodule into a malignant one. Another explanation is that the histological findings of follicular carcinoma (capsular and/or vascular invasion) may occur only when a nodule grows beyond a certain size. The situation is different for PTC, in which malignancy is histologically determined based upon nuclear and cellular morphological changes, and such results can be valid in thyroid nodules regardless of their size.

The variation in the results of the studies dealing with the relationship between nodule size and malignancy risk might be related to the differences in selection criteria. In most of these studies, a relationship between nodule size and risk of malignancy was valid, particularly for patients with follicular or Hurthle cell neoplasm. Tuttle and cols. examined 103 patients for whom cytological findings were consistent with a follicular neoplasm and who had undergone surgery at some point during a 5-year period. Final pathological results showed that the rate of malignancy was 40% and 13% for nodules ≥ 4 cm and < 4 cm, respectively (p = 0.03) (30). In another study including 243 patients with a follicular adenoma and 152 patients with a follicular carcinoma, carcinomas were more likely to be ≥ 3 cm when compared to adenomas (53% *vs.* 34%, p < 0.001). Based on these findings, the authors recommended near total or total thyroidectomy for patients with an FNAB consistent with a follicular neoplasm and a nodule size ≥ 3 cm (31). Chen and cols. examined 37 patients with Hurthle cell adenoma and 20 patients with Hurthle cell carcinoma with a mean tumor size of 2.4 ± 0.2 cm and 4.0 ± 0.4 cm, respectively. The rate of malignancy was 17% for tumors ≤ 1 cm, 23% for tumors 1-4 cm and 65% for tumors ≥ 4 cm. Depending on these results, they recommended near total or total thyroidectomy for all patients with a Hurthle cell neoplasm ≥ 4 cm (32).

The most important limitation of our study was its retrospective design. The study group was composed of patients who had undergone surgery, which probably caused increased malignancy rates. In our series, larger nodules were prompted to surgery, even when FNAB was benign, owing to fear of malignancy potential or other compression symptoms or substernal expansions. In addition, the patients in whom surgery was indicated for toxic multinodular goiter were also included in our series. These patients had also undergone surgery due to larger nodule dimensions, despite low risk of malignancy.

In conclusion, nodule size does not appear to be an independent risk factor for malignancy in thyroid nodules. Such an inference is valid for both nodule diameter and volume. For follicular and Hurthle cell carcinomas, the risk of malignancy seems to increase linearly when nodule size increases. Although malignant nodules had significantly higher volume compared to benign ones among nodules ≥ 4 cm, an optimal cutoff value could not be determined. Our results suggest that it is not possible to predict malignancy or decide on surgical resection considering only the diameter or volume of the nodule.
